# A bibliometrics and visualization analysis of cannabidiol research from 2004 to 2021

**DOI:** 10.3389/fphar.2022.969883

**Published:** 2022-11-04

**Authors:** Liu Liu, Jianxing Liu, Ming Zhao, Meiming Cai, Fanzhang Lei, Xiaofeng Zeng, Bofeng Zhu

**Affiliations:** ^1^ Guangzhou Key Laboratory of Forensic Multi-Omics for Precision Identification, School of Forensic Medicine, Southern Medical University, Guangzhou, China; ^2^ School of Forensic Medicine, Kunming Medical University, Kunming, China

**Keywords:** cannabidiol, bibliometrics analysis, data visualization, research hotspots, co-citation analysis, burst detection

## Abstract

Cannabidiol, a non-psychoactive component extracted from the plant cannabis sativa, has gained growing focus in recent years since its extensive pharmacology effects have been founded. The purpose of this study intends to reveal the hot spots and frontiers of cannabidiol research using bibliometrics and data visualization methods. A total of 3,555 publications with 106,793 citations from 2004 to 2021 related to cannabidiol were retrieved in the Web of Science database, and the co-authorships, research categories, keyword burst, and reference citations in the cannabidiol field were analyzed and visualized by VOSviewer and Citespace software. Great importance has been attached to the pharmacology or pharmacy values of cannabidiol, especially in the treatment of neuropsychiatric disorders, such as epilepsy, anxiety, and schizophrenia. The mechanisms or targets of the cannabidiol have attracted the extreme interest of the researchers, a variety of receptors including cannabinoids type 1, cannabinoids type 2, 5-hydroxytriptamine1A, and G protein-coupled receptor 55 were involved in the pharmacology effects of cannabidiol. Moreover, the latest developed topic has focused on the positive effects of cannabidiol on substance use disorders. In conclusion, this study reveals the development and transformation of knowledge structures and research hotspots in the cannabidiol field from a bibliometrics perspective, exploring the possible directions of future research.

## Introduction

Cannabidiol (CBD), one of the major components of cannabis sativa without psychological dependence (Devinsky et al., 2014), has presented great pharmacology values in neuropsychiatric disorders due to its anti-inflammation and anti-oxidation effects (Campos et al., 2016; Melas et al., 2021; Scarante et al., 2021). A recent double-blind study revealed that cannabidiol decreased the convulsive-seizure frequency in the Dravet syndrome (a complex childhood epilepsy disorder) (Devinsky et al., 2017). In June 2018, the US Food and Drug Administration (FDA) approved the first CBD-based drug, Epidiolex (GW Pharmaceuticals, England) for the treatment of seizures associated with Dravet syndrome and Lennox-Gastaut syndrome (VanDolah et al., 2019). CBD alleviated Alzheimer’s disease (AD)-related neuron damage by regulating inflammation and oxidative stress (Vallée et al., 2017). Preclinical and clinical trials proved the therapeutic effects of CBD on Parkinson’s disease (PD)-related non-motor symptoms (Crippa et al., 2019). CBD could also be used as a candidate drug for the treatment of pain (Xiong et al., 2012; De Gregorio et al., 2019). In addition, the pharmacology values of CBD in substance abuse (Lee et al., 2017), anti-tumor (Sultan, 2018), and multiple sclerosis (Mecha et al., 2013) have been investigated in recent years. Another deeply researched topic is the molecular mechanism of CBD in these disorders, which may be related to the endocannabinoid system (ECS), serotonin system, transient receptor potential vanilloid (TRPV) channels, G protein-coupled receptor 55 (GPR55), peroxisome proliferator-activated receptor-gamma (PPARγ), and so on (Campos et al., 2012). Although great research progress has been made in CBD research, the hot spots and frontiers still need to be illustrated to provide useful information for future research directions.

Bibliometrics is a discipline that explores the distribution structure, quantitative relationship, and variation trend of literature using mathematics and statistics methods (Yin et al., 2021). Bibliometrics and data visualization could provide an overview of research categories or themes, co-authorships, keywords frequencies, and most cited articles or journals, which contribute significantly to revealing the hot spots and frontiers in one specific area (Song et al., 2021; Chu et al., 2022).

In this study, we performed the bibliometrics analysis based on the published literature related to CBD research from 2004 to 2021 in the Web of Science (WOS) database, and several networks of categories, keywords, co-authorships, and co-cited references were visualized by VOSviewer and Citespace software. This study aims to better understand the dynamic changes of current CBD research and explore the possible directions for future research.

## Materials and methods

### Data searching

The literature published in the WOS core collection Science Citation Index Expanded (SCI-E) database from 2004 to 2021 was collected on 15 March 2022. Searching strategies used in this study were as follows: the topic was set as “cannabidiol”, the literature types were “article” and “review”, and the language was “English” only. Therefore, a dataset consisting of 3,555 publications was exported for subsequent analysis.

### Bibliometrics analysis and data visualization

The annual number of publications and citations, and the top 15 most productive countries were visualized in Microsoft Excel version 2019 based on the report of WOS. A world map was generated using Bibliometrix *R* package version 3.2.1 to reveal the geographical distribution of publications. A pie chart was drawn based on WOS categories using Excel. The bibliometrics analysis and data visualization in this study were performed by Citespace version 5.8.R3 and VOSviewer version 1.6.18 software. Citespace could simplify the search for significant papers in a specific area so that one can search for visually salient features, including categories, co-cited references, cluster information, and brust detection (Chen, 2004; Chen, 2006). VOSviewer was used here to perform the networks of co-authorship and keyword co-occurrence.

## Results

### General analysis

**FIGURE 1:**
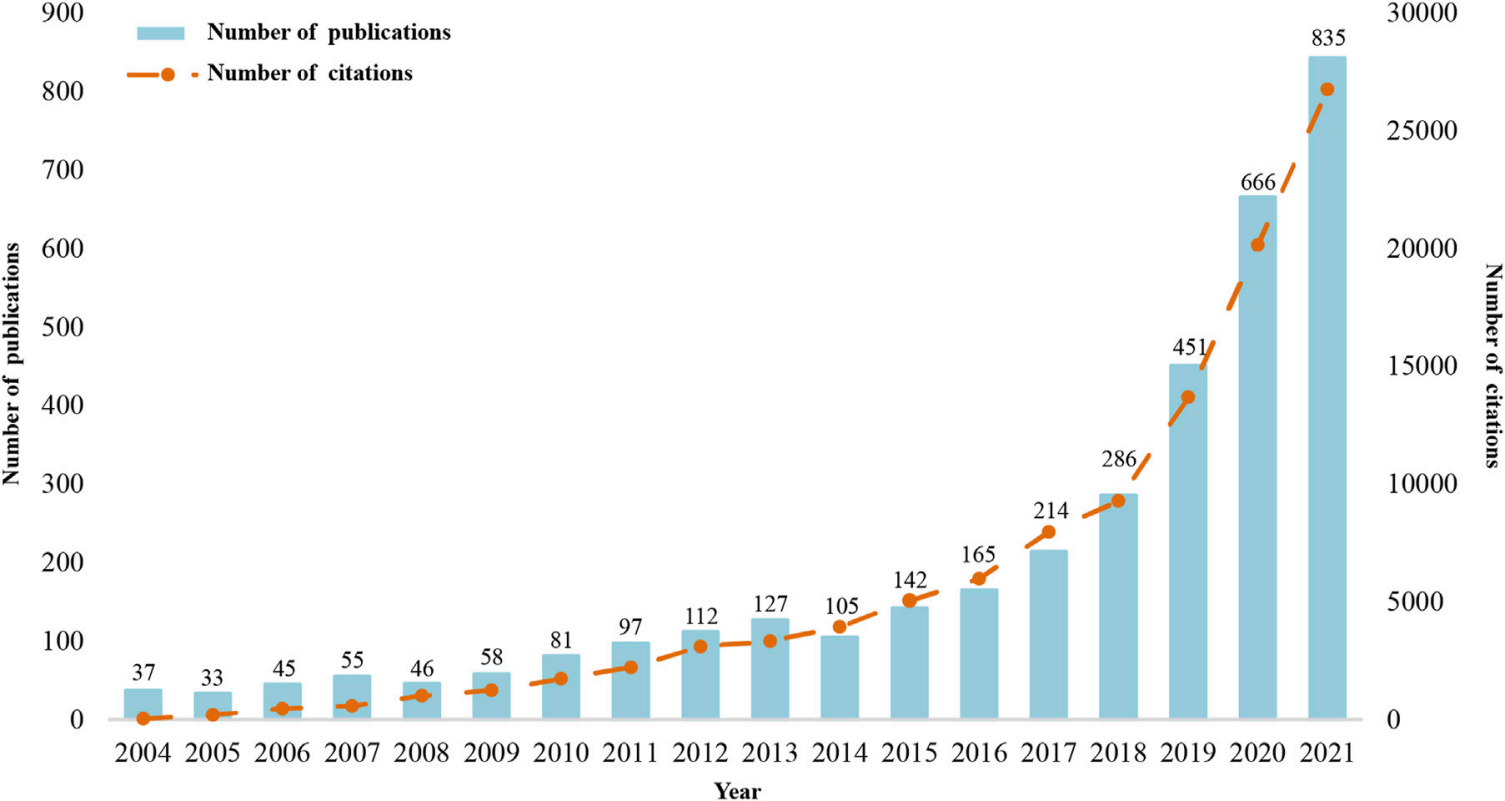
Annual number of publications and citations.

A total of 3555 publications including 2,719 articles (76.48%) and 836 reviews (23.52%) from 2004 to 2021 were retrieved in the WOS SCI-E database, with a sum of 106,793 citations, each of the publications was cited on average 30.04. Figure 1 showed the variation of publications and citations with the years. The annual number of publications increased year by year, except for 2005, 2008 and 2014, and the annual growth of publications exceeded 100 for the first time in 2019. The number of citations per year has grown rapidly from 2019 to 2021. We also retrieve the publications related to CBD that were published in English before 2004 (data not shown). A total of five articles were published with a sum of 74 citations, the first of which was published in 1988 with 11 citations. One of the 5 was divided into pharmacology and pharmacy by WOS, and the rest belongs to chemistry fields.

**FIGURE 2:**
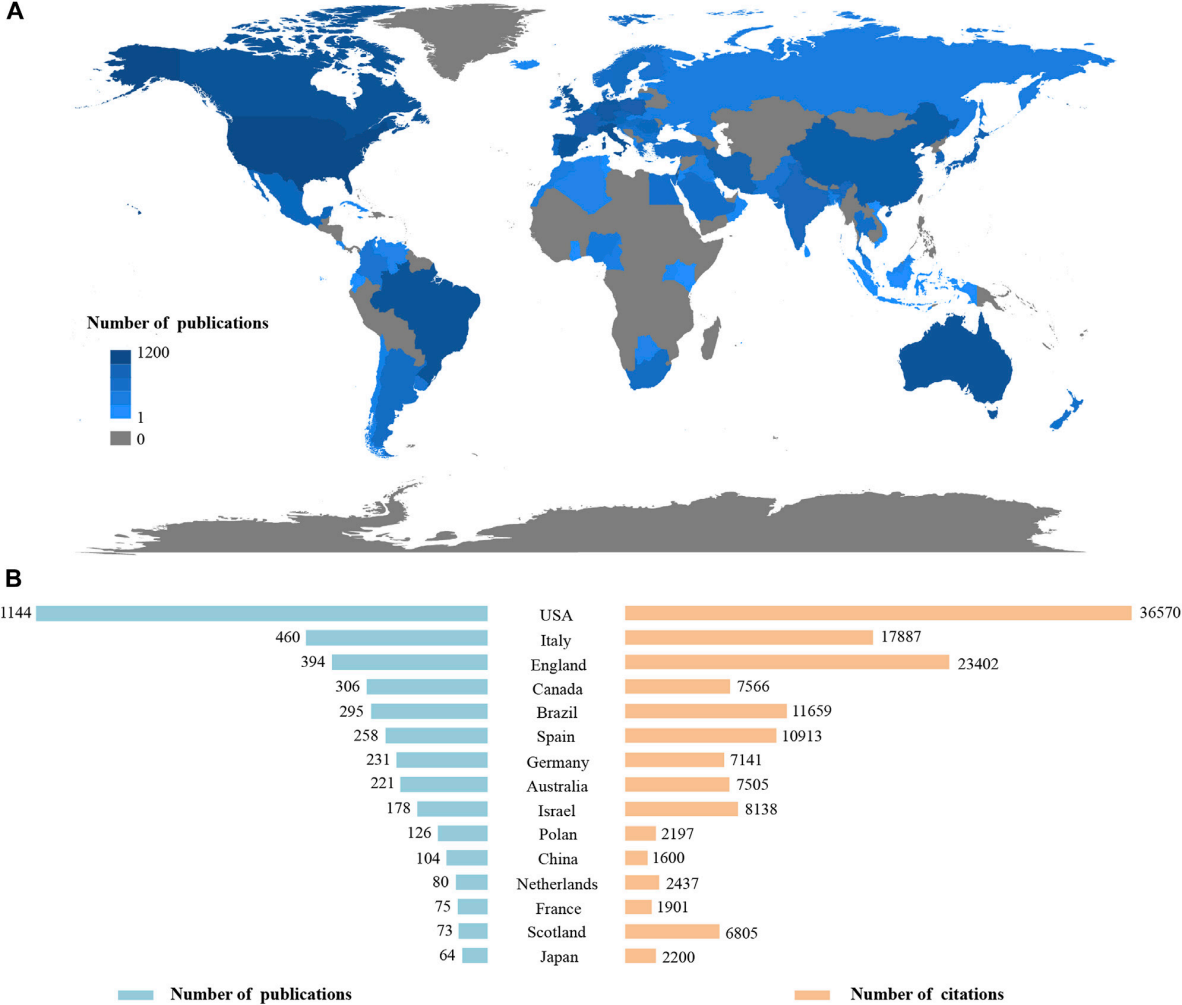
**(A)** Geographical distribution of publications. **(B)** The top 15 most productive countries in the publications and citations.

During this period, a total of 94 countries have published literature related to CBD research. Figure 2A displayed the geographical distribution of publications, and the top 15 most productive countries with their number of publications and citations were shown in Figure 2B. The United States contributed the highest to this topic, publishing 1,144 papers and being cited 36,570 times. The second most productive country was Italy, with 460 publications and 17,887 citations. England published 394 papers with 23,402 citations, an average citations of 59.40 per paper. Notably, Scotland showed the highest average number of citations, each of the literature was cited on average 93.22.

### Category analysis

**FIGURE 3:**
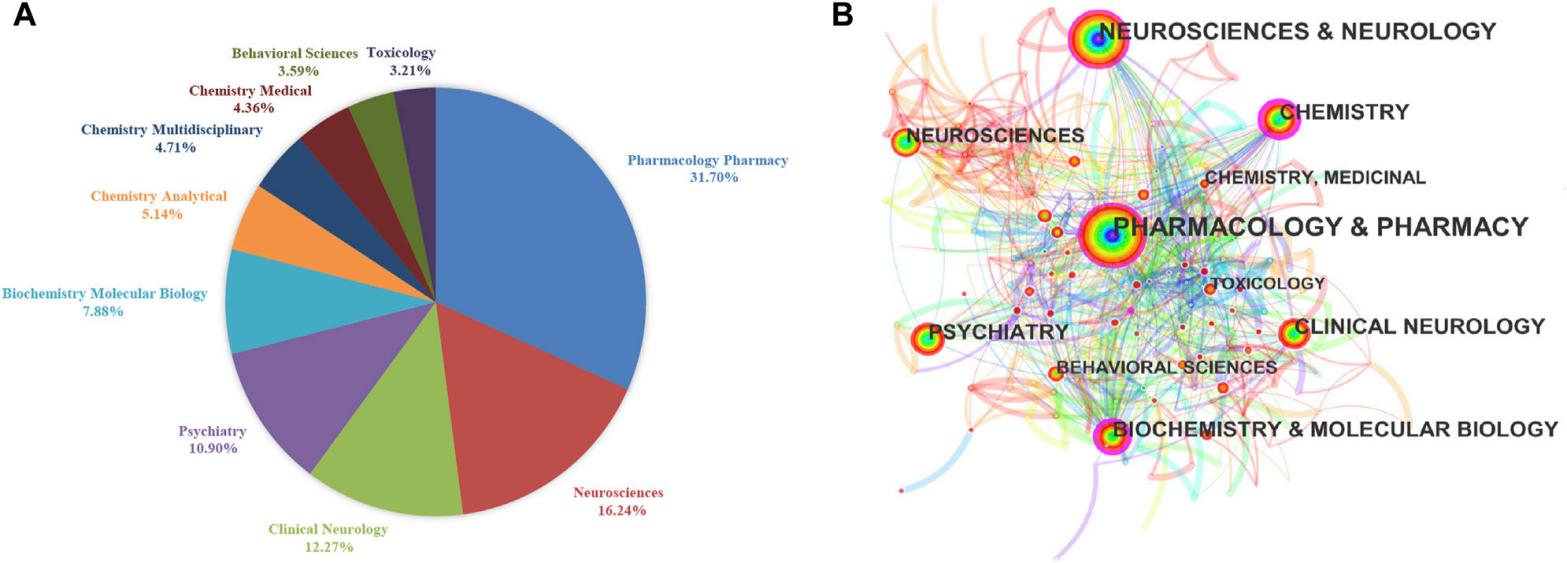
**(A)** The top 10 most published categories. **(B)** The network of the top 10 most published categories.

The category of publications is an important indicator to reveal the hot spots and applications of CBD research. Figure 3A presented the top 10 most published categories in WOS SCI-E database. There are a total of 1271 publications divided into Pharmacology/Pharmacy, which account for 31.70% of the top 10 most published categories. Neurosciences is the second largest published category, with 651 publications (16.24%), followed by Clinical Neurology with 492 publications (12.17%), Psychiatry with 437 publications (10.90%), and Biochemistry Molecular Biology with 316 publications (7.88%). Subsequently, we performed the co-occurrence network of categories based on the dataset of WOS using Citespace software. 248 nodes and 1190 links were included in the network, and Figure 3B showed the top 10 most published categories. Each of the nodes represents a specific category, and the node size was used here to reflect the frequency of category occurrence. Betweenness centrality provides a method to quantify the importance of the node’s position in a network (Chen, 2006), and the nodes with purple trims indicate the high betweenness centrality. Figure 3B revealed that Pharmacology & Pharmacy was the highest co-occurrence category in the network, and its centrality was 0.14. Moreover, Chemistry presented the maximum centrality (0.26), suggesting that Chemistry is a pivotal point or tipping point in the network.

### Co-authorship analysis

**FIGURE 4:**
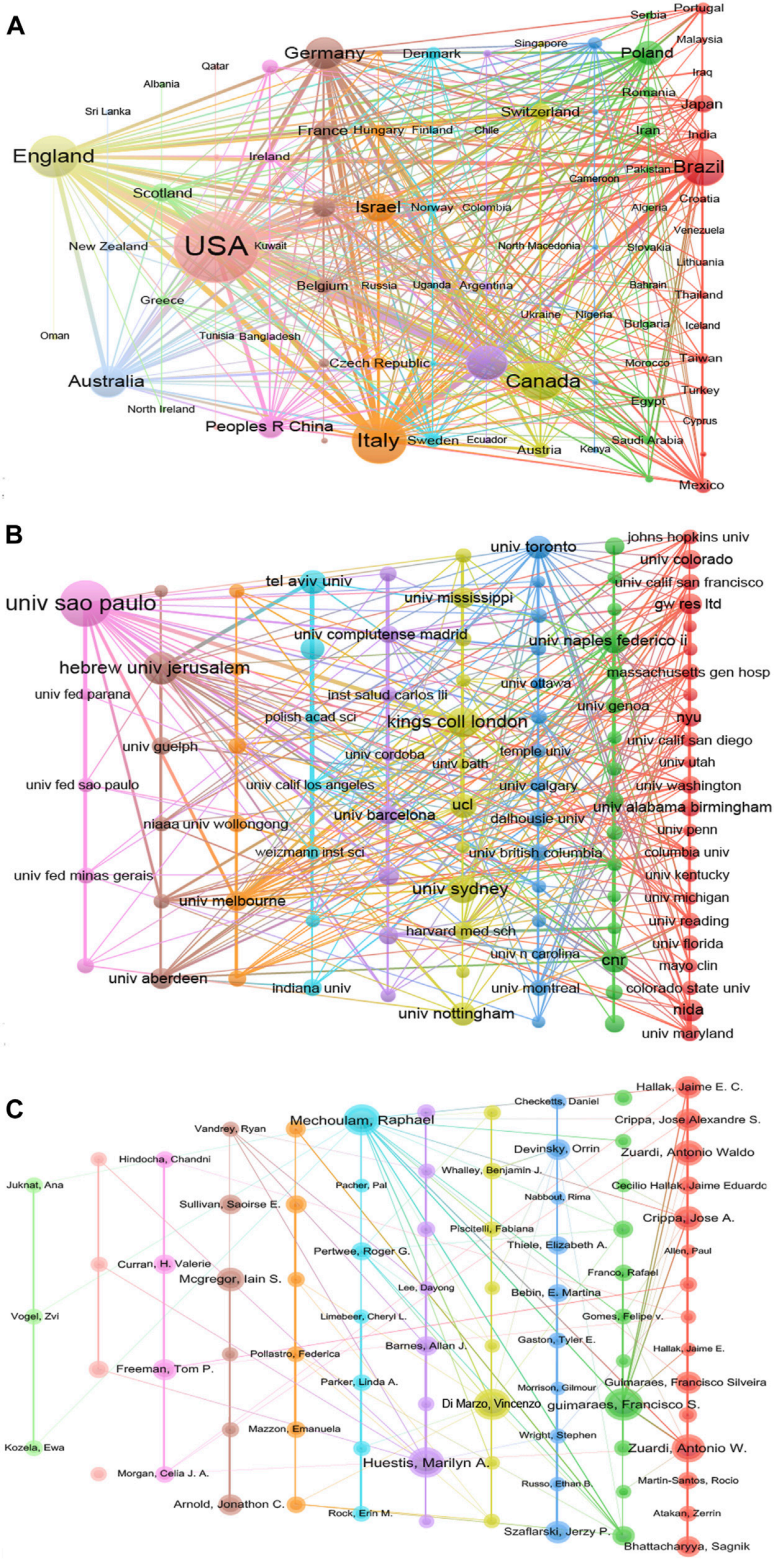
Co-authorships analyses. **(A)** Co-authorships between different countries. **(B)** Co-authorships between different institutions. **(C)** Co-authorships between different authors.

To investigate the co-authorship between different countries, institutions, and authors, we performed the collaboration network based on the WOS dataset using VOSviewer software. There are a total of 94 countries, 3,569 institutions, and 14,040 authors who have contributed to this topic. As shown in Figure 4, each node represents a different country/institution/author, the node size represents the number of documents, the thickness of connecting lines represents the strength of inter collaboration, and each color represents a cluster. Figure 4A presented the inter collaboration between different countries. Some of the 94 countries in the network were not connected, and the largest set consisted of 86 countries. The United States was the most productive and cooperative country, with 573 link strengths. The second most cooperative country was England, with a sum of 412 link strengths, and the third-highest number of documents. Italy, Spain, Canada, Germany, and Brazil also showed high productivity and cooperative strength in the network.

Figure 4B displayed the inter collaboration between different institutions. We picked out the top 100 most productive institutions to generate the partnership network, but two institutions were excluded due to a lack of cooperation with other institutions. The University of Sao Paulo was the most productive and cited institution, with the highest cooperative link strength. The University of Sao Paulo was the first and the largest modern comprehensive university in Brazil. It is also the most important scientific research center in Brazil. Table 1 submitted detailed information on the top 10 most cooperative institutions. Nine of the top 10 most cooperative institutions were from different research universities and only one was owned by a pharmaceutical company, indicating that the current study on CBD was still dominated by basic research. GW Res Ltd. established a world-leading position in the development of plant-derived cannabinoid therapeutics, which has developed the first FDA-approved CBD-based drug, Epidiolex for the treatment of Dravet syndrome and Lennox-Gastaut syndrome.

**TABLE 1:** The top 10 most collaborative institutions.

Institution	Documents	Citations	Country	Total collaborative strength
University of Sao Paulo	208	10,140	Brazil	134
King’s College London	90	5126	England	108
Hebrew University of Jerusalem	105	6412	Israel	88
GW Research Limited	42	2266	England	67
University of Toronto	52	1259	Canada	65
University of Melbourne	39	2718	Australia	63
University College London	54	2934	England	57
New York University	37	3946	United States	55
University of Naples Federico II	54	3540	Italy	54
Tel Aviv University	56	1818	Israel	46

Figure 4C showed the inter collaboration between different authors. 106 authors met the threshold of 10 or more published articles, while only 86 authors were founded to connect with the other authors in the network. The most productive author was Guimaraes FS with a total of 51 documents, 2,406 citations, and 65 link strengths. However, the most cited author was Di Marzo V with a sum of 4,419 citations, 47 documents, and 53 link strengths. The most cooperative author was Huestis MA with a total of 98 link strengths, 45 documents, and 1,684 citations.

### Funding agency contribution

The source of funding reflects, to a certain extent, the different levels of the country’s support for the field at different stages and the enthusiasm of the research community. Table 2 presented the top 10 most productive funding agencies for CBD research. United States Department of Health and Human Services was listed in the first, providing funds for 426 documents, which accounts for 11.98% of all the publications. The followed funding agencies were the National Institutes of Health (NIH)-USA and the European Commission with 422 and 360 documents, accounting for 11.87% and 10.13% of all the publications, respectively. Three of the top 10 most productive funding agencies were from the United States and sponsored a total of 1,071 publications, which accounts for 30.12% of all the publications. Additionally, the residual top 10 most productive funding agencies including three Brazil agencies, two England agencies, and one Canada agency, accounting for 12.19%, 4.81%, and 1.97% of all the publications, respectively.

**TABLE 2:** The top 10 most productive funding agencies in the cannabidiol field.

Organization	Documents	Country	Percentages (%)
United States Department of Health & Human Services	426	United States	11.98
National Institutes of Health (NIH) - United States	422	United States	11.87
European Commission	360	European Commission	10.13
NIH National Institute on Drug Abuse (NIDA)	223	United States	6.27
Conselho Nacional de Desenvolvimento Cientifico E Tecnologico (CNPQ)	199	Brazil	5.60
Fundacao de Amparo a Pesquisa do Estado de Sao Paulo (FAPESP)	130	Brazil	3.66
Coordenacao de Aperfeicoamento de Pessoal de Nivel Superior (CAPES)	104	Brazil	2.93
United Kingdom Research and Innovation (UKRI)	92	England	2.59
Medical Research Council United Kingdom (MRC)	79	England	2.22
Natural Sciences and Engineering Research Council of Canada (NSERC)	70	Canada	1.97

### Journal contribution

A total of 3,555 publications were published in 999 different journals. Table 3 listed the top 10 most-cited journals in the CBD area. The most cited journal was the *British Journal of Pharmacology* with cited times of 9,906, and a sum of 95 papers were published. *Epilepsia* was listed second with 2,212 citations and 42 papers, followed by *Neuropsychopharmacology* with 2,093 citations and 21 papers. Four of the 10 listed journals were from England, other four journals were from the United States, and the rest were from Germany and Switzerlands. The top three journals with the highest impact factor (IF) were the *British Journal of Pharmacology* (8.739), *Neuropsychopharmacology* (7.885), and *Epilepsia* (5.866). In addition, the H-index and ISSN of the journal were also listed in Table 3.

**TABLE 3:** The top 10 most-cited journals in the cannabidiol field.

Jorunal	Country	If (2020)	Documents	Citations	H-index	ISSN
British Journal of Pharmacology	England	8.739	95	9906	211	0007–1188
Epilepsia	United States	5.866	42	2212	191	0013–9580
Neuropsychopharmacology	England	7.855	21	2093	219	0893–133X
Psychopharmacology	Germany	4.53	43	1959	196	0033–3158
Journal of Pharmacology and Experimental Therapeutics	United States	4.03	25	1952	225	0022–3565
Journal of Psychopharmacology	England	4.153	34	1834	114	0269–8811
Epilepsy and Behavior	United States	2.937	67	1797	104	1525–5050
Cannabis and Cannabinoid Research	United States	5.8	96	1601	17	2578–5125
Frontiers in Pharmacology	Switzerland	5.811	78	1581	81	1663–9812
Neuropharmacology	England	5.251	45	1572	167	0028–3908

### Keywords co-occurrence and burst detection

**FIGURE 5:**
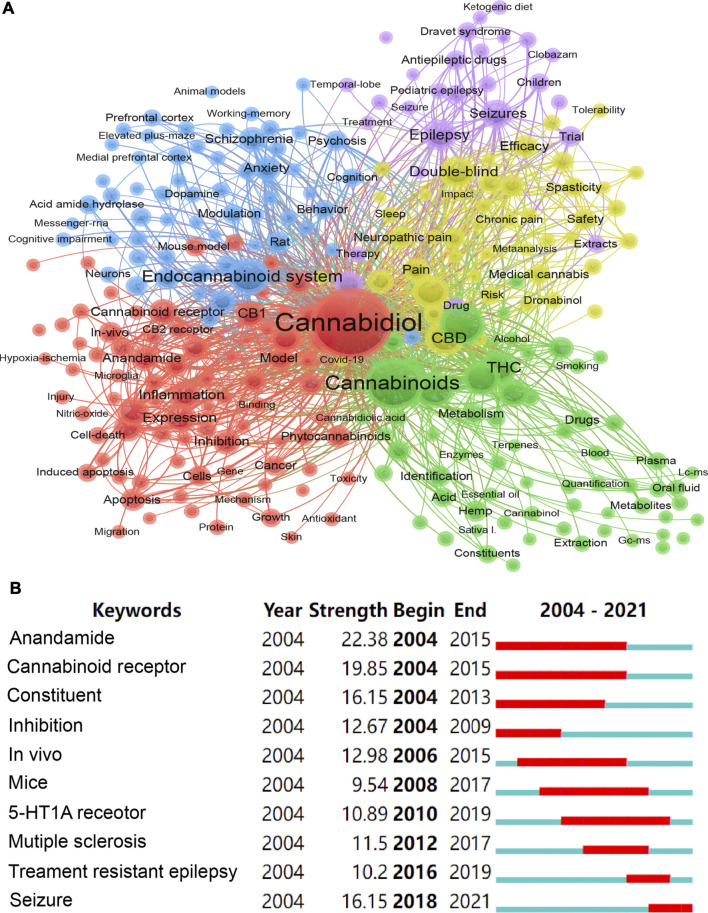
Keywords analyses. **(A)** The network of the keywords co-occurrence and clustering. **(B)** The top 10 keywords with the strongest bursts.

The keyword plays an important role to highlight and emphasize the focus and core content of the whole article. The keyword co-occurrence network enables an overview of the core content of published articles, as well as reveals the connections between the content. There are a total of 11,116 keywords included in the 3555 publications, and 282 keywords meeting the frequency threshold of more than 20. Figure 5A displayed the co-occurrence network consisting of 282 keywords using the Vosviewer software. Each node represents a keyword, the node size indicates the frequency of the keyword, and each color reveals a cluster in the network. The top 15 keywords with the highest occurrence frequencies and strongest connection were cannabidiol (1,960, 11,444), cannabinoids (949, 5,747), cannabis (535, 3,336), endocannabinoid system (405, 2,750), delta (9)-tetrahydrocannabinol (394, 2,770), CBD (363, 2,393), marijuana (348, 2,236), THC (326, 2,275), delta-9-tetrahydrocannabinol (303, 2,204), and double-blind (282, 2,024), cannabinoid (248, 1,565), epilepsy (240, 1,565), expression (233, 1,376), *in-vitro* (214, 1,385), seizures (213, 1,316). 282 keywords in the network were divided into five clusters, and the detailed information was summarized as follows: 1) Mechanism was represented by red: cannabinoid receptor, CB1, CB2, inflammation, antioxidant, protein, and apoptosis; 2) Disease was represented by blue: schizophrenia, anxiety, psychosis, behavior, rat, prefrontal cortex, and cognitive impairment; 3) Treatment of epilepsy was represented by purple: epilepsy, Dravet syndrome, therapy, seizures, antiepileptic drugs, and children; 4) Clinical trial was represented by yellow: double-blind, neuropathic pain, efficacy, medical cannabis, safety, risk, and tolerability; 5) Synthesis and metabolism were represented by green: bioavailability, blood, plasma, chromatography, metabolism, pharmacokinetics, synthetic cannabinoids, extraction, identification, and quantification.

Brust detection served to recognize the emergent terms and sharp increases of interest (Chen, 2006), which significantly contributed to revealing the frontiers in one specific field. We performed the keyword burst detection based on the dataset of WOS using Citespace software, and the top 10 keywords with the strongest bursts were displayed in Figure 5B. Anandamide was the top 1 strongest burst keyword that emerged in 2004 and ended in 2015, revealing that anandamide was extensively studied and discussed during this period. The seizure was the most recent keyword with high burst strength that appeared in 2018. The bursts of the keyword cannabinoid receptor, constituent, *in vivo*, mice, and 5-ht1a receptor sustained at least 10 years.

### Reference co-citation analysis and timeline map

Reference citation and co-citation frequencies could clearly illustrate the intellectual concerns in a field. In general, an article with a high co-citation frequency suggests it has a high citation frequency. Table 4 listed the top 10 most cited articles from 2004 to 2021 in the CBD field. The most cited reference was published by Ryberg et al. (2007), which revealed that the orphan receptor GPR55 was a novel cannabinoid receptor. A review published by Pertwee (2008) summarized the diverse CB1 and CB2 receptor pharmacology of three plant cannabinoids, indicating that CBD displayed high potency as a CB2 receptor antagonist. The reference published by Russo (2011) reviewed the pharmacology effects of CBD in anti-oxidant, anti-anxiety, anti-depressant, anti-convulsant, and so on, and the possible mechanisms were also discussed. Devinsky et al. (2017) conducted a double-blind trial of cannabidiol for drug-resistant seizures in 120 individuals, revealing that CBD exhibited greater effects in decreasing the convulsive-seizure frequency than placebo. This research was published in the *New England Journal of Medicine* with the highest H-index (1030) in the top 10 most cited articles. Moreover, Leweke et al. (2012) demonstrated that CBD alleviated the psychotic symptoms of schizophrenia by enhancing anandamide signaling. Mechoulam and Parker (2013) summarized the actions of the endocannabinoid system on anxiety, depression, neurogenesis, reward, cognition, learning, and memory. Notably, three of the top 10 most cited articles attached concerns to the pharmacology effects of CBD on seizures, and these articles were published by the team of Devinsky et al. (2014), Devinsky et al. (2016), Devinsky et al. (2017), another three articles focus on the mechanisms and molecular targets of CBD pharmacology effects (Ryberg et al., 2007; Pertwee, 2008; Mechoulam and Parker, 2013).

**TABLE 4:** The top 10 most cited articles from 2004 to 2021.

Title	Authors	Journal	Citations	IF (2020)	H-index
The orphan receptor GPR55 is a novel cannabinoid receptor	Ryberg, E, et al.	British Journal of Pharmacology	984	8.739	211
The diverse CB1 and CB2 receptor pharmacology of three plant cannabinoids: Delta (9)-tetrahydrocannabinol, cannabidiol and Delta (9)-tetrahydrocannabivarin	Pertwee, RG.	British Journal of Pharmacology	955	8.739	211
Taming THC: potential cannabis synergy and phytocannabinoid-terpenoid entourage effects	Russo, Ethan B, et al.	British Journal of Pharmacology	664	8.739	211
Trial of Cannabidiol for Drug-Resistant Seizures in the Dravet Syndrome	Devinsky, O, et al.	New England Journal of Medicine	628	91.253	1030
Cannabidiol enhances anandamide signaling and alleviates psychotic symptoms of schizophrenia	Leweke, FM, et al.	Translational Psychiatry	547	6.222	82
The Endocannabinoid System and the Brain	Mechoulam, R, et al.	Annual Review of Psychology	528	24.137	243
Non-psychotropic plant cannabinoids: new therapeutic opportunities from an ancient herb	Izzo, AA, et al.	Trends in Pharmacological Sciences	491	14.819	218
Cannabidiol: Pharmacology and potential therapeutic role in epilepsy and other neuropsychiatric disorders	Devinsky, O, et al.	Epilepsia	459	5.866	191
Changes in Cannabis Potency Over the Last 2 Decades (1995–2014): Analysis of Current Data in the United States	ElSohly, MA, et al.	Biological Psychiatry	450	13.382	319
Cannabidiol in patients with treatment-resistant epilepsy: an open-label interventional trial	Devinsky, O, et al.	Lancet Neurology	444	44.182	291

**FIGURE 6:**
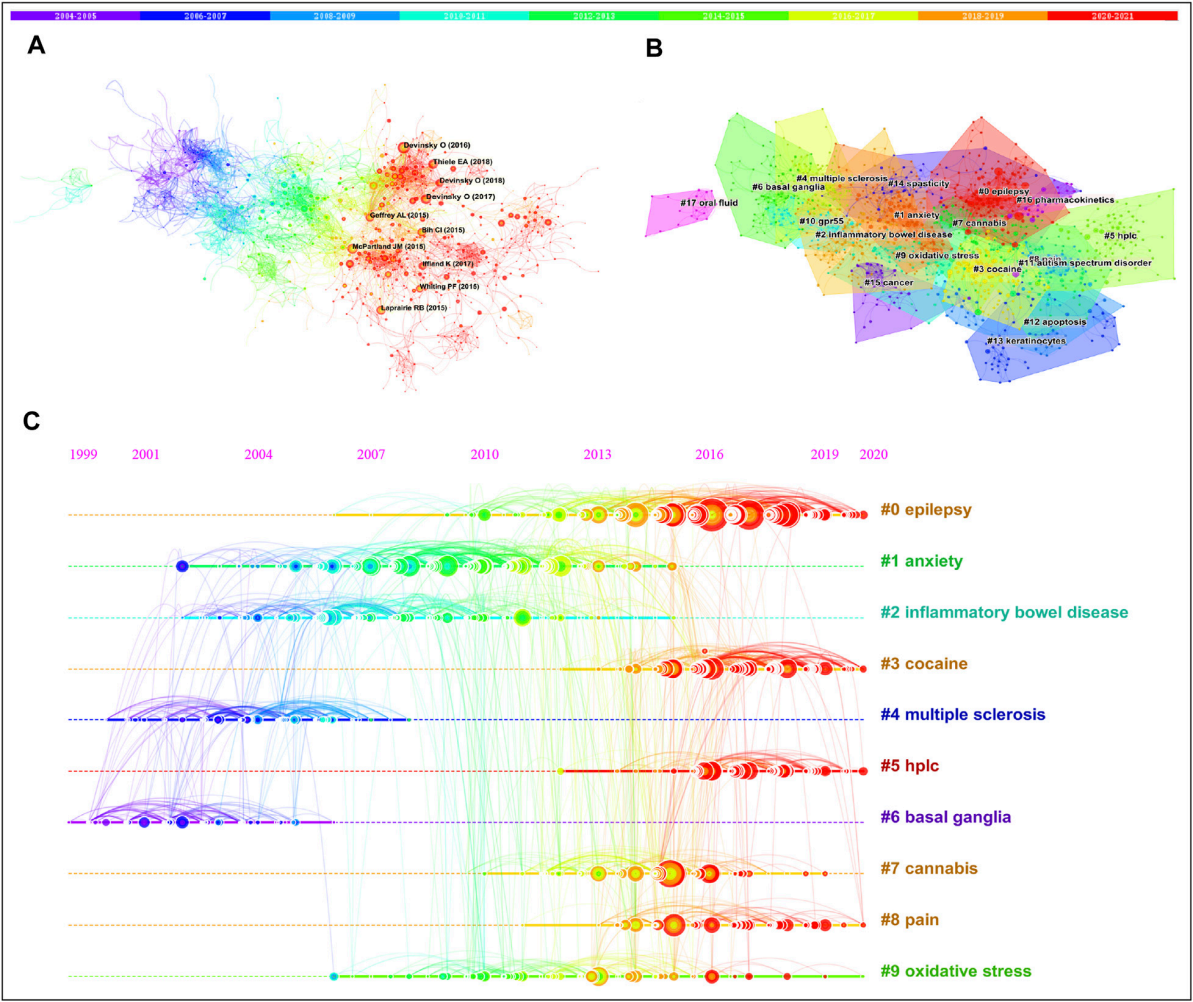
Reference co-citation analyses. **(A)** The network of the co-cited articles. **(B)** Cluster analysis of the co-cited articles. **(C)** A timeline view of the top 10 clusters.

Next, we performed the co-citation network of reference based on the WOS dataset using the Citespace software. 1,248 nodes and 6,077 links were founded in Figure 6A, and the top 10 most co-cited articles were highlighted in the network. The top 2 most co-cited articles were published by Devinsky et al. (2016), Devinsky et al. (2017), with 354 and 288 co-citations, respectively. The former article assessed the safety, tolerability and efficacy of CBD in patients already receiving stable doses of antiepileptic drugs, indicating that CBD might decrease the frequency of seizure frequency with sufficient safety in children and young adults with highly treatment-resistant epilepsy (Devinsky et al., 2016). The latter focused on drug-resistant epilepsy in Dravet syndrome and evaluated the efficacy and safety of CBD in it, suggesting that cannabidiol reduced the frequency of convulsive seizures in children and young adults with Dravet syndrome but was associated with adverse events including somnolence and elevation of liver enzyme levels (Devinsky et al., 2017). The followed most co-cited articles were published by Thiele et al. (2018) with 238 co-citations, and Devinsky et al. (2018) with 222 co-citations, both of them explored the effects of CBD on seizures in the Lennox–Gastaut syndrome through the clinical trial. Laprairie et al. (2015) illustrated that Cannabidiol is a non-competitive negative allosteric modulator of CB1 receptors. Besides, the side effects, toxicity, and drug-drug interaction of CBD therapies were also discussed based on clinical data and animal studies (Geffrey et al., 2015; Whiting et al., 2015; Iffland and Grotenhermen, 2017). In summary, the clinical trials of CBD on seizures have absorbed great interest from researchers, and investigated the mechanism of the CBD effect helps to better elucidate the pharmacology value of CBD. In addition, there are serious concerns about the issues of safety and side effects of CBD, more trials still need to be conducted for solving these problems.

Clusters of co-cited articles provided a method to detect the transition of research frontiers in an area (Chen, 2006). Figure 6B displayed the clusters of co-cited articles using Citespace software. The modularity Q and silhouette values of the clusters were 0.791 and 0.9081, respectively, which indicated the structure of the clusters was significant and all of the clusters were convincing. All of the co-cited articles were assigned to 18 different clusters, and the clusters of the same color represent co-citations made within the same time slice. The first cluster of co-cited articles was epilepsy, and the followed were anxiety, inflammatory bowel disease, cocaine, multiple sclerosis, HPLC, basal ganglia, cannabis, and oxidative stress. Figure 6C provided a timeline view of the top 10 clusters, and detailed information about these clusters was listed in Table 5. The early studies focused on the pharmacology effects of CBD on multiple sclerosis (2004), inflammatory bowel disease (2008), and anxiety (2009), as well as the regulatory effect of CBD on the endocannabinoid system and the serotonin system in the basal ganglia (2002). In 2012, the anti-oxidative effects of CBD attracted the attention of researchers. In 2014, researchers were interested in exploring the values of cannabis. Notably, the effects of CBD in the treatment of epilepsy have been enthusiastically investigated in 2016, and great progress has also been made in drug development. The most recent themes in the CBD area were cocaine and HPLC. CBD could be used as a potential therapeutic drug for the treatment of cocaine use disorders (Mahmud et al., 2017; Luján et al., 2020).

**TABLE 5:** The detailed information of 10 clusters in Figure 5C.

Cluster ID	Size	Silhouette	Mean (year)	Top terms
0	155	0.937	2016	Epilepsy
1	146	0.844	2009	Anxiety
2	124	0.867	2008	Inflammatory bowel disease
3	116	0.889	2017	Cocaine
4	90	0.899	2004	Multiple sclerosis
5	84	0.96	2017	Hplc
6	76	0.889	2002	Basal ganglia
7	71	0.879	2014	Cannabis
8	70	0.943	2016	Pain
9	68	0.876	2012	Oxidative stress

### Burst detection of co-cited reference

**FIGURE 7:**
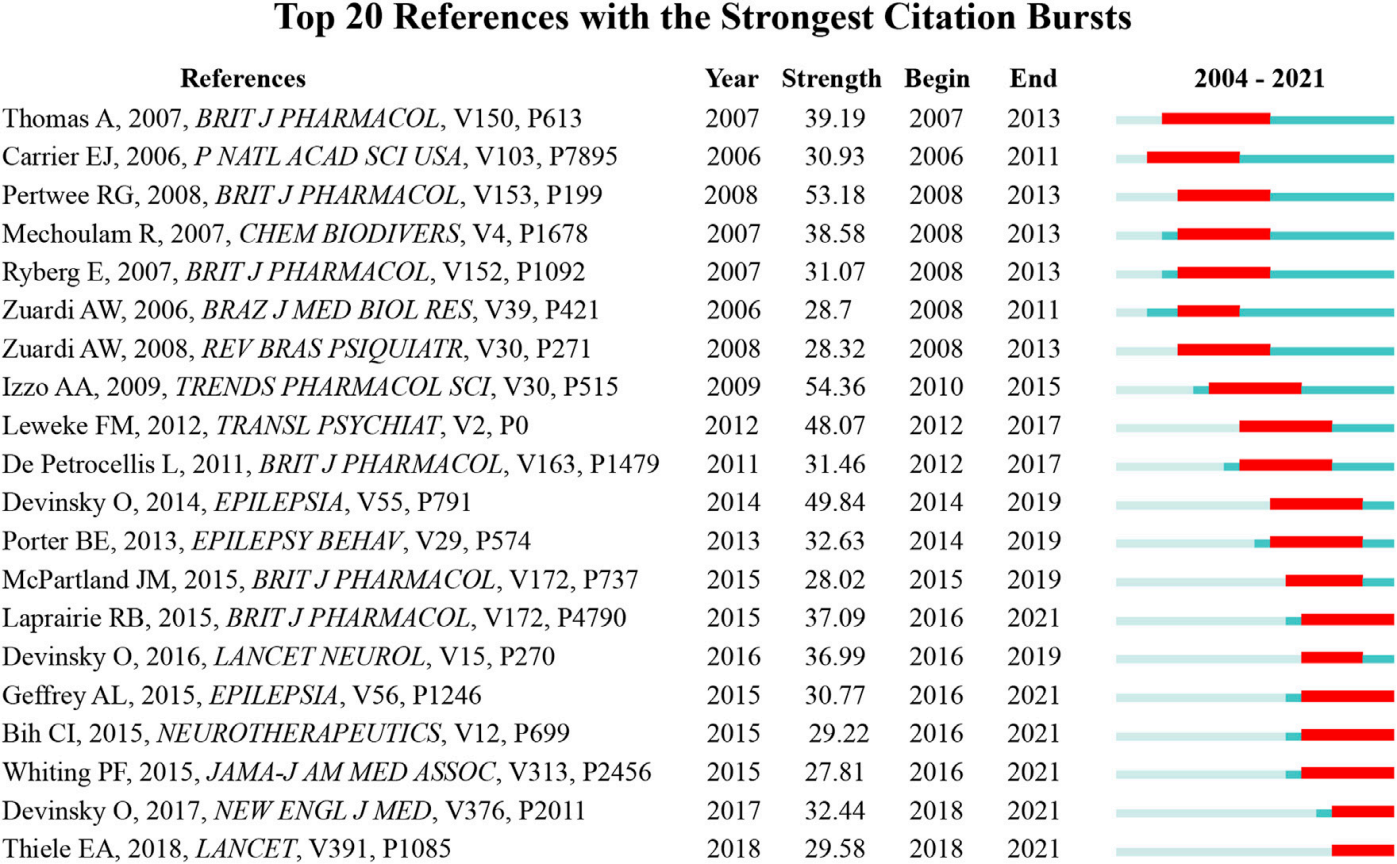
The top 20 references with the strongest citation bursts.

Figure 7 presented the top 20 references with the strongest citation bursts. The article with the highest burst strengths was entitled “Non-psychotropic plant cannabinoids: new therapeutic opportunities from an ancient herb” (Izzo et al., 2009), which reviewed the therapeutic applications of CBD in inflammation, diabetes, cancer, affective and neurodegenerative diseases. The article with the earliest citation bursts has begun in 2006 and was entitled “Inhibition of an equilibrative nucleoside transporter by cannabidiol: A mechanism of cannabinoid immunosuppression” (Carrier et al., 2006), which demonstrated that CBD could decrease inflammation by enhancing adenosine signaling. The latest burst articles were “Trial of cannabidiol for drug-resistant seizures in the Dravet syndrome” (Devinsky et al., 2017) and “Cannabidiol in patients with seizures associated with Lennox-Gastaut syndrome (GWPCARE4): a randomised, double-blind, placebo-controlled phase 3 trial” (Thiele et al., 2018), both of them were focused on the clinical trials of CBD in the seizures related Dravet syndrome or Lennox-Gastaut syndrome. According to Figure 7, the themes of the top 20 burst references consisted of the molecular mechanism investigation (7), clinical trial on epilepsy (6), pharmacology effects (6), and treatment of schizophrenia (1).

## Discussion

Our present study attempts to reveal the hot spots and research trends in the CBD field, a dataset including 3555 publications from 2004 to 2021 was analyzed using a bibliometrics method. Based on the searching reports of the WOS database, we firstly evaluated the publication and citation trends over the years and the contributions of different counties/funding agencies. The research categories, the collaboration relationships, keywords bursts, and co-cited references were detected by bibliometrics tools.

The number of published articles related to CBD has increased over time except for 2005, 2008, and 2014, and significant growth could be observed from 2019 to 2021. Moreover, the number of citations showed growth continually, especially from 2019 to 2021, indicating increased focus in the field among researchers in recent years. In 2017 and 2018, great concerns were attached to the clinical trials of CBD in epilepsy. With the FDA approval of the first CBD-based drug for the treatment of seizures associated symptoms in 2018, the therapeutic values of CBD in neuropsychiatric disorders have been increasingly recognized by researchers.

Among the 94 countries that have participated in CBD research, the United States was the world leader with 1,114 articles and a total of 36,570 citations, which could be explained by two possible reasons. Firstly, the financial policies of the funding agencies in the field. The United States Department of Health and Human Services, National Institutes of Health (NIH)-USA, and NIH National Institute on Drug Abuse (NIDA) have provided funds for a total of 1,071 publications, which accounts for 30.12% of all the publications. Secondly, the collaboration strength with other countries. The United States was the most cooperative country with other countries, and a total of 573 link strengths could be detected. Still, there is only one United States institution on the list of the top 10 most cooperative institutions. One possible reason is that there are many institutions working in this area in the United States, but they do not have outstanding advantages. Another possible reason has to do with United States funding policies. Fund management departments need to allocate funds to more institutions, but this argument needs more evidence to back it up. England contributed second in citations in the field, with 394 articles cited 23,402 times, which was related to the predominant research institutions, such as King’s College London, GW Research Limited, and University College London. Notably, GW Pharmaceuticals has sponsored several impactful clinical trials, which have contributed significantly to the drug development of seizure treatment (Devinsky et al., 2016, 2017; Thiele et al., 2018). On the other hand, England had the second strongest collaboration with other countries, and 412 link strengths were included in the network. In addition, Italy, Canada, and Brazil have also been pivotal in the advancement of the field, due to the high outputs and citations of their articles, and extensive partnership with other countries. In Brazil, the University of Sao Paulo was the most productive institute with 208 documents published, accounting for 70.5% of all the documents. The University of Sao Paulo was also the most collaborative institute with other countries in the CBD field. Furthermore, Brazil accounted for three of the top 10 most productive funding agencies, which indirectly reflected that the Brazil government attaches great importance to CBD research.

Co-authorships analysis revealed the most productive, most cited, and extensive cooperative authors in the CBD field. The top three most productive authors were Guimaraes FS, Mechoulam R, and Di Marzo V, they were from three different institutions, the University of Sao Paulo (Brazil), Hebrew University of the Jerusalem (Israel), and Laval University (Canada). The top three authors with the highest citations were Di Marzo V, Mechoulam R, and Devinsky O (New York University, United States). While the top three authors who had the most corporations with others were completely different from the top three most productive or cited authors. Huestis MA was the most cooperative author with others in the field and was from Jefferson University (United States), the followed authors were Zuardi AW (University of Sao Paulo, Brazil), and Crippa JA (University of Sao Paulo, Brazil).

The category analysis of 3555 publications suggested that Pharmacology/Pharmacy, and Neuroscience/Neurology were the hot researched subjects in the CBD field, and Chemistry plays a key role in the evolution of research subjects. The cluster analysis of keywords divided 282 keywords with a frequency greater than 20 times into five clusters, which could be concluded as the following five themes: 1) Mechanism; 2) Disease; 3) Treatment of epilepsy; 4) Clinical trial; 5) Synthesis and metabolism. Keywords bursts detection indicated that seizure was one of the research hot spots in the field. Over the past decade, the pharmacology effects of CBD on neuropsychiatric disorders have been widely explored. It is worth noting that great achievements were made in the treatment of seizures, a CBD-based drug, Epidiolex, was approved by the FDA in 2018. However, with the Epidiolex used in the clinical trials, a series of side effects, including somnolence, decreased appetite, diarrhea, rash, sleep disorder, infections, and so on have raised concerns of researchers (Taylor et al., 2018; Pauli et al., 2020). To better elucidate the long-term efficacy, safety, and tolerability of CBD in the treatment of epilepsy, large-scale or in-depth clinical trials and analyses are necessary. In Parkinson’s disease, clinical trials indicated that CBD could alleviate the rapid eye movement sleep behaviour disorder (Chagas et al., 2014a), anxiety symptoms (de Faria et al., 2020), and improve the life quality of PD patients (Chagas et al., 2014b). In schizophrenia, CBD showed beneficial effects for anti-psychotic symptoms and improved cognitive performance in patients through anandamide-independent and dopamine-independent mechanisms (McGuire et al., 2018; Leweke et al., 2021). In conclusion, the promising research and application prospects of CBD in neuropsychiatric disorders have been confirmed, and its neuroprotective effect may be an important direction for future drug development of CBD. For the treatment of multiple sclerosis-related symptoms, such as spasticity and pain, a drug combined delta (9)-tetrahydrocannabinol (THC) and CBD, Sativex has been investigated by researchers (Wade et al., 2004; Rog et al., 2005). In 2020, Sativex (GW Pharmaceuticals, England) has been approved by more than 25 countries in the world.

As revealed in the reference co-citation analysis and burst detection, researchers attached great importance to pharmacology effects of CBD in the alleviation of seizures related symptoms. The reference with the highest co-citations illustrated the clinical trials of CBD on epilepsy, and six of the top 20 burst references focused on epilepsy. Notably, the molecular mechanisms, targets, or signaling pathways of the CBD effects were also the hot spots in the field. ECS is involved in modulating the developments and functions of the brain and thus plays an important role in depression, anxiety, cognitive, memory, and rewarding effects (Mechoulam and Parker, 2013; Micale et al., 2013). CB1 and CB2 are the two major receptors of the ESC, but there are still divergences regarding the regulatory role of CBD on these receptors. One view supported that CBD has a low affinity or even no activity for CB1 and CB2 receptors (Ryberg et al., 2007; McPartland et al., 2015). In contrast, another perspective pointed out that CBD was a high-efficiency antagonist of CB1 and CB2 receptor agonists (Thomas et al., 2007; Laprairie et al., 2015). 5-HT1A receptor was one of the top 10 bursts keywords, which has been thoroughly studied in the CBD field. CBD presented effects on antiepileptic, antianxiety, and antidepressant disorders by activating the 5-HT1A receptor (Campos and Guimarães, 2008; Zanelati et al., 2010; Devinsky et al., 2014). GPR55 is a high affinity receptor of the cannabinoids family, and CBD can be activated as a selective antagonist of GPR55 to prevent inflammation-associated impairments (Li et al., 2013; Chiurchiù et al., 2015). In addition, PPARγ has also displayed significant value in the anti-inflammatory and antioxidative effects of CBD (Vallée et al., 2017; Sonego, 2018). TRPV channels are the potential targets for CBD activity, especially TRPV2 medicated Ca2+ dynamics (Nabissi et al., 2013; Hassan et al., 2014). The modulatory effects of CBD on TRPV channels may be involved in anti-neuroinflammation (Hassan et al., 2014), anticancer (Santoni et al., 2020), and antinociception (Maione et al., 2011). The above researches indicates that the anti-inflammation effect of CBD may be one of the core pharmacological mechanisms of CBD. In fact, by targeting inflammation, CBD decreased the AD-related and PD-related neuron damage (Vallée et al., 2017, 2021), preventing multiple sclerosis-associated inflammatory impairments (Mecha et al., 2013) and alcohol-induced liver injury (Wang et al., 2017), and inhibiting methamphetamine-induced reinstatement in rats (Karimi-Haghighi et al., 2020). Additionally, the anti-inflammation effect of CBD was mediated by several signaling pathways, including GPR55, PPARγ, and TRPV channels. Do these signaling pathways exhibit time-specificity and spatial-specificity under the same conditions? Whether these signaling pathways interact with each other or are co-regulated by a specific signaling molecule? These are worthy of deeper consideration and exploration in the future.

The timeline view of the co-cited references revealed the effect of CBD on cocaine use disorders is a newly developed theme in recent years. Studies have reported that CBD effectively attenuated the cocaine-induced rewarding effects, drug-seeking behaviors (Luján et al., 2020; Ledesma et al., 2021), and seizures (Gobira, 2015) in the preclinical trials. A double-blind trial indicated that CBD reduced cue-induced craving and anxiety in heroin use disorders (Hurd et al., 2019). Moreover, the great value of CBD in the treatment of methamphetamine (Karimi-Haghighi and Haghparast, 2018), morphine (Rodríguez-Muñoz et al., 2018), and alcohol (Turna et al., 2019) use disorders have also been proved. In short, these studies provided new perspectives and approaches in treating substance use disorders, however, more researches and efforts are still needed before using CBD as a therapeutic drug.

## Conclusion

Our present study performed a bibliometrics analysis in the CBD field based on the literature published from 2004 to 2021 with the expectation to reveal the research hot spots and frontiers. The pharmacology and pharmacy of CBD have always been enthusiastically investigated by researchers, particularly in neuropsychiatric disorders, such as epilepsy, schizophrenia, anxiety, etc. CBD meditated the CB1, CB2, 5-HT1A, GPR55, PPARγ receptors, and TRPV channels may be explained its extensive pharmacology effects. In recent years, the values of CBD in the treatment of substance use disorders have attracted researchers’ interest.

## Data Availability

The original contributions presented in the study are included in the article/Supplementary Materials, further inquiries can be directed to the corresponding authors.
